# A standardized approach for measuring multivariate equity in vaccination coverage, cost-of-illness, and health outcomes: Evidence from the Vaccine Economics Research for Sustainability & Equity (VERSE) project

**DOI:** 10.1016/j.socscimed.2022.114979

**Published:** 2022-06

**Authors:** Bryan Patenaude, Deborah Odihi, Salin Sriudomporn, Joshua Mak, Elizabeth Watts, Gatien de Broucker

**Affiliations:** a Johns Hopkins Bloomberg School of Public Health, Department of International Health and International Vaccine Access Center (IVAC), USA; b University of Minnesota School of Public Health, Department of Health Policy & Management, USA

## Abstract

Following a call from the World Health Organization in 2017 for a methodology to monitor immunization coverage equity in line with the 2030 Agenda for Sustainable Development, this study outlines a standardized approach for measuring multivariate equity in vaccine coverage, economic impact, and health outcomes. The Vaccine Economics Research for Sustainability & Equity (VERSE) composite vaccination equity measurement approach is derived from literature on the measurement of socioeconomic inequality combined with measures of direct unfairness in healthcare access. The final metrics take the form of a concentration index for vaccination coverage where individuals are ranked by multivariate unfairness in access and an absolute equity gap representing the difference in coverage between the top and bottom quintiles of individuals ranked by multivariate unfairness in access. Regression decomposition is applied to the concentration index to determine each factor's relative influence on observed inequity. These methods are applied to India's National Family Health Survey (NFHS) from 2015 to 2016 to assess the equity in being fully-immunized for age vaccination coverage and zero-dose status. The multivariate absolute equity gap is 0.120 (SE: 003) and 0.371 (SE: 0.008) for zero-dose status and fully-immunized for age, respectively. Therefore, the most disadvantaged quintile is 12 percentage points more likely to be zero-dose than the most advantaged quintile and 37.1 percentage points less likely to be fully immunized. The primary correlate of unfair disadvantage for both outcomes is maternal education accounting for 27.4% and 19.1% of observed inequality. The VERSE model provides a standardized approach for measuring multivariate vaccine coverage equity. It also allows policymakers to determine the relative magnitude of factors influencing multivariate equity rather than only the correlates of socioeconomic or bivariate equity. This framework could be adapted to track equitable progress toward Universal Health Coverage (UHC) or outcomes beyond the vaccine space.

## Highlights

• This study outlines a standardized approach and toolkit for measuring multivariate inequality in vaccination coverage.
• The model isolates unfair from fair correlates of inequality in coverage.
• Application to India shows maternal education is the factor most associated with vaccine inequity.
• Greater inequality exists for being fully-immunized for age than being zero-dose in India.

## 1 Introduction

Following a call from the World Health Organization (WHO) in 2017 for a methodology to monitor immunization coverage equity in line with the 2030 Agenda for Sustainable Development, the Vaccine Economics Research for Sustainability & Equity (VERSE) project proposes a standardized approach and novel toolkit for measuring and tracking multivariate vaccine-related equity in coverage, economic impact, and health outcomes (Arsenault et al., 2016; United Nations General Assembly, 2015). The methodology of the VERSE project, presented in this paper, builds upon existing equity methodologies and toolkits, such as the WHO Health Equity Assessment Toolkit (HEAT) (Hosseinpoor et al., 2016, 2018), by expanding the outcomes assessed and providing a standardized approach for aggregation across multiple factors influencing equity including socioeconomic, demographic, educational, sex-based, and geospatial covariates to generate composite equity metrics that are trackable over time and comparable between settings.

The composite equity metrics produced by the VERSE model help address several important shortcomings of commonly applied equity metrics and toolkits in vaccine research, including the ability to combine several factors influencing equity into one indicator, the ability to determine the relative degree to which factors contribute toward overall observed equity, the ability to separate fair and unfair factors influencing equity, and the ability to compare equity across several types of vaccine-related outcomes (Galston, 2021; Pressman et al., 2021). Since equity is typically multivariate in composition, an over-emphasis on any one dimension over which equity is measured, such as income or wealth, will hide persistent inequality along dimensions not perfectly correlated with income (Alonge and Peters, 2015).

In response to an increasing push for a multivariate expansion to the assessment of socioeconomic inequality, numerous governmental institutions, and international organizations, including the European Commission (European Commission, 2021), the United States Census Bureau (Glassman, 2019), the government of the United Kingdom (Vizard and Speed, 2015) and the United Nations (McKnight, 2018), have begun expanding beyond a singular focus on income or wealth as the basis for measuring and tracking social equity. However, in examining equity in healthcare access, research on inequality typically focuses on one factor or a series of separate bivariate assessments (Papageorge et al., 2021; Millar et al., 2021; Portnoy et al., 2020). While this type of sub-group comparison over specific factors is commonplace, a systematic approach for combining and measuring the composite inequality over multiple groups remains lacking, particularly in the context of vaccination (Bosch- Capblanch et al., 2017). The VERSE model (and its derived toolkit) addresses the need for a standardized multivariate approach to equity measurement of healthcare access in a way that can both be applied to specific interventions, such as vaccines, and to overall dimensions of universal health coverage, including population reached, services provided, and financial risk protection afforded.

In addition to laying out a methodology for the standardized multivariate evaluation of vaccine coverage equity, this paper applies the methodology and toolkit to assess the level of equity in vaccination coverage and zero-dose vaccination status utilizing the National Family Health Survey (NFHS-IV) in India as a case study. Gavi, the Vaccine Alliance defines zero-dose children as children who have not received a single dose of any of the four major routine pediatric vaccinations, including vaccines for diphtheria, pertussis, and tetanus (DTP), measles-containing vaccine (MCV), and polio vaccine (OPV or IPV) (Cata-Preta et al., 2021). In addition to the measurement of inequity in zero-dose status of children in India, the VERSE tool provides decomposition to highlight the relative weight of factors influencing inequity in the composite concentration index-based metric and compares how each state performs on dimensions of coverage and multivariate equity using a coverage-equity plane. In addition to the more rigorous composite equity concentration index, an absolute equity gap (AEG) metric is also produced due to its more intuitive interpretation for policy-makers non-technical audiences.

## 2 Methods

The primary VERSE composite vaccination equity assessment metrics are a multivariate concentration index and absolute equity gap measure derived from literature on the measurement of socioeconomic equity by Wagstaff and Erreygers combined with measures of direct unfairness in healthcare access outlined in the works of Fleurbaey, Schokkaert, Cookson, and Barbosa (Alonge and Peters, 2015; Barbosa and Cookson, 2019; Williams and Cookson, 2006; Fleurbaey and Schokkaert, 2011; Wagstaff, 2011; Fleurbaey and Schokkaert, 2009; O’ Donnell et al., 2007). The composite concentration index metric takes the form of a concentration index for vaccination coverage where, instead of ranking individuals by income, individuals are ranked by a multivariate unfair disadvantage in access. Unfair disadvantage as parameterized in the VERSE model is an adaptation of a direct unfairness measure. It computes the predicted vaccination coverage from a logistic regression model (for binary outcomes) or a generalized linear model (for continuous outcomes) as a function of multiple factors contributing to fair and unfair sources of variation in vaccination coverage (Fleurbaey and Schokkaert, 2011). Fair sources of variation in coverage include whether the child is underage to receive the vaccine according to the national immunization schedule of the country or countries examined. Unfair sources of variation included in the standard model are the sex of the child, maternal education level, socioeconomic status derived from a wealth index, coverage by health insurance, urban or rural designation, and geopolitical subunit of residence. These factors were chosen based on near-universal data collection through typical demographic and health surveys (DHS) and other nationally representative health surveys (DHS, 2021). Data permitting as appropriate to the setting, the ranking model may be expanded to include other readily available unfair dimensions contributing to access, including racial or ethnic group, caste, religious affiliation, or age.

**Fig. 1:**
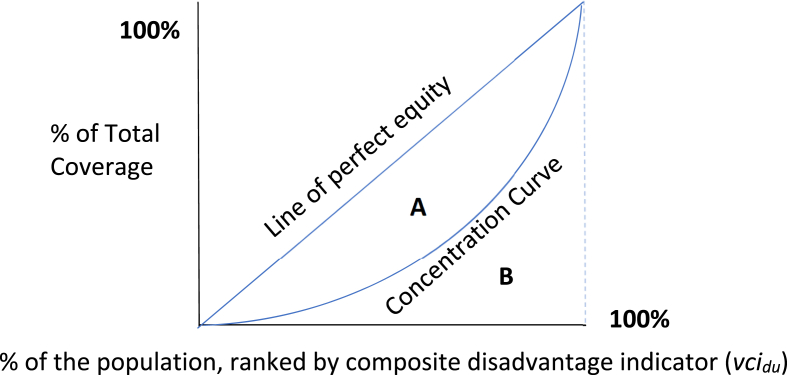
Concentration index illustrative example.

Additionally, preference-based factors influencing inequality, including the exhibition of vaccine hesitancy or need-based factors, such as allergy to vaccination, may be incorporated into the model to improve the isolation of unfair sources contributing to differences in vaccine access. The direct unfairness ranking metric is then assessed as the predicted probability of vaccination, holding the fair determinants at reference levels and allowing the unfair determinants to vary. For continuous variables, the predicted value of the continuous output holds the fair determinants at reference levels and allows the unfair determinants to vary. This unfair disadvantage metric is then utilized as the ranking variable to compute a concentration index, alongside the cumulative share of the outcome, which produces the primary composite coverage equity metric (Fig. 1).

For the binary case of vaccination coverage where the outcome takes a value of 1 if the child received the vaccine and 0; otherwise, the direct unfairness metric for vaccination coverage indicator (*vci*_*du*_) can be written as:

$$vci_{du}=vci_{predicted}\left(N_{ref},P_{ref},Z_{i},X_{ref}\right)$$

where:

***N*** = vector of need variables (in the vaccine case, only the age of the child is used)
***P*** = vector of preference for healthcare variables
***Z*** = vector of unfair variables (e.g., socioeconomic status, urban/rural, sex of the child, maternal education)
***X*** = vector of neither fair nor unfair variables (e.g., variables that may confound the relationship between unfair predictors and coverage)
*vci*_*predicted*_*=* Predicted probability of receiving care holding need (*N*) & neutral (*X*) variables at reference levels

For the VERSE model, the basic assumption is that there are no neutral variables and that parent preferences for vaccination are either not observable or are a function of the ***Z*** vector variables (e.g., maternal education) and therefore should be counted as unfair sources of variation and not true preferences. As such, the direct unfairness in vaccination coverage indicator (vci_du_) can be simplified as:

$$vci_{du}=vci_{predicted}\left(N_{ref},Z_{i}\right)$$

Therefore, under the logistic framework letting vaccination status (*v)* = 1 if vaccinated and 0 otherwise, the predicted *vci*_*du*_ for individual *i* can be written as:

$$\text{Let}\;p_{i}=\text{Pr}\left(v=1|N_{ref},Z_{i}\right)$$

$$\text{logit}\left(p_{i}\right)=\alpha +\beta Z_{i}+\gamma N_{ref}+\varepsilon$$

Using this setup, the predicted value for individual *i* is defined by: $\hat{p_{i}}$ = *vci*_*du*_*.*

Once *vci*_*du*_ is obtained, it is then used as the ranking variable to compute either a Wagstaff or Erreygers modified concentration index, replacing the more traditional ranking variable of socioeconomic status (Wagstaff, 2011; O’ Donnell et al., 2007). As such, the predicted probability of vaccination conditional on unfair determinants (*vci*_*du*_), or in the continuous case the predicted healthcare access level based on unfair determinants, functions in the same manner as a wealth index creating a scale where the relative rank of individuals over (*vci*_*du*_) depicts their degree of relative unfair disadvantage in obtaining the outcome in question. This is utilized alongside the cumulative share of attainment of the health outcome to compute a concentration-style index, which exhibits the properties of a Gini-index, being bounded between −1 and 1 and, therefore, is standardized by construction.

The VERSE toolkit enables the production of either a traditional Wagstaff concentration index ($CI_{W}$) of the form:

$$CI_{W}=\frac{2Cov(vci_{direct},F\left(vci_{du}\right))}{\mu vc}$$

where:

$vci_{direct}=$ Directly standardized individual level of healthcare (observed vaccination coverage)
$F\left(vci_{du}\right)=$ The cumulative distribution function of direct unfairness
μvc= Mean level of healthcare (vaccination coverage) across the entire population

Alternatively, the Erreygers modified concentration index $\left(CI_{E}\right)$ calculated from the Wagstaff metric as:

$$CI_{E}=4*\mu vc*CI_{W}$$

While not produced in the base application of the VERSE tool, it is also possible to produce the Horizontal Inequity Index (HII) from the toolkit, following the same overall approach utilizing the form:

$$HII=CI-CI_{fair-determinant-predict}=\frac{2Cov(vci_{observed},F(vci_{du})}{\mu vc}-\frac{2\;Cov(vci_{fair},F(hvi_{du})}{\mu vc_{fair}}$$

where:

$$vci_{fair}=vci_{predicted}\left(N_{i},Z_{ref}\right)$$

And *vci*_*fair*_ is modeled through a logistic regression (for the binary outcome of vaccination status) where:

$$\text{Let}\;p_{i}=\text{Pr}\left(v=1|N_{i},Z_{ref}\right)$$

$$\text{logit}\left(p_{i}\right)=\alpha +\beta Z_{ref}+\gamma i_{f}+\varepsilon$$

Using this model, the predicted values can be expressed as: $\hat{p_{i}}$ = *vci*_*fair*_*.*

Finally, to aid in the interpretation of the absolute level of inequity observed, the VERSE model produces a second absolute equity gap (AEG) metric which is of the form:

$$AEG=vci_{observed}\left(top\;20\%\;\left(F\left(vci_{du}\right)\right)\right)-vci_{observed}\left(Bottom\;20\%\;(F\left(vci_{du}\right)\right))$$

For ease of interpretation, the primary outcome recommended for interpreting and conveying the VERSE toolkit results to policymakers is the AEG. The AEG is directly interpreted as the absolute difference between the vaccination outcome (*vci*) achieved by the top 20% and bottom 20% of the population, ranked by unfair disadvantage. Mathematically, this is equivalent to isolating the top and bottom quintiles from the Lorenz curve used to estimate the Wagstaff (direct) concentration index (CI).

Since the composite concentration index produced by the VERSE model is based on a traditional concentration index, regression-based Kitagawa-Blinder-Oaxaca decomposition can be employed to generate the cumulative share of overall observed inequality relating to each of the fair and unfair factors (Barbosa and Cookson, 2019; Blinder, 1973; Oaxaca, 1973). Like with other types of concentration indices, a decomposition is necessary for every separate concentration index generated to translate changes in the metric into changes in the variation accounted for by each of the factors. Even across different vaccines, two concentration indices for two different vaccines may both equal to 0.1 but decompose into very different allocations across dimensions (e.g., maternal education, wealth, sex of the child).

A fundamental assumption of the VERSE model is that every child should be vaccinated by the recommended age in the national immunization schedule. As such, the only source of fair variation in vaccination status should be the *age of the child*. This means that children younger than the recommended age for a specific vaccine can fairly be expected not to have received a vaccination and should be netted out of the unfair disadvantage metric computation process. All other sources of variation in vaccination status (socioeconomic status, sex of the child, maternal education, caste, urban/rural designation, state/district, and health insurance coverage) should be considered unfair sources of variation in vaccination status. For other healthcare outcomes, the designation of fair sources of variation should be driven by the empirical evidence in the literature or expert consensus.

Moving beyond health access outcomes: vaccine coverage can be replaced with modeled outcomes such as DALYs averted from vaccination, cost-of-Illness averted from vaccination, or out-of-pocket expenditure incurred, respectively, to compute multivariate inequity over financial risk protection and health outcomes. Continuous outcomes employ a generalized linear regression model to predict the unfair outcome attainment and utilize the relative rank of unfair disadvantage in outcomes as the ranking criteria in lieu of the predicted probability of attaining the outcome utilized in the binary case. For the continuous case, the y-axis of the concentration curve becomes the cumulative share of outcome attainment while the x-axis is the cumulative rank of unfair disadvantage. For economic outcomes, such as out-of-pocket expenditure or cost-of-illness, facility type (e.g., public or private) should be included as fair, preference-based measures and adjusted for when ranking individuals by unfair disadvantage in those outcomes. Each of the resulting multivariate concentration indices can be decomposed to determine the percent contribution of each determinant to overall composite inequity. The resulting analysis can be conducted separately for individual vaccines as well as over a coverage indicator for zero-dose, fully-immunized for age, or completed the entire routine vaccination schedule by 24 months of age.

**Fig. 2:**
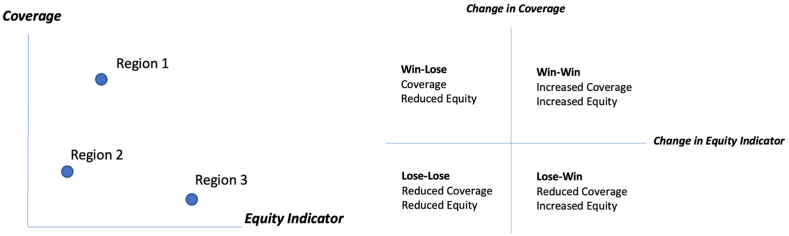
Equity coverage plane illustrative examples.

Finally, the VERSE toolkit permits the examination of equity-coverage trade-offs through the presentation of an equity-coverage plane, which pairs the composite concentration index metric with a coverage metric to examine the relative performance of sub-national geographic units on the dual goals of equity and efficiency. This graphic can be utilized to compare the performance on each dimension at a single point in time or centered around a baseline measure to showcase the direction of movement between baseline and endline (Fig. 2).

## 3 Results

Data for the case-study application of the VERSE toolkit's methodology to India utilizes the fourth round of the National Family Health Survey (NFHS-IV) conducted in 2015–2016 (Government of India MOH Family and Welfare, 2017). Two binary outcomes are defined using the survey, zero-dose status is defined as not having received a single dose of diphtheria, pertussis, and tetanus (DTP) vaccine, measles-containing vaccine (MCV), or polio vaccine (OPV or IPV) by 24 months of age (Cata-Preta et al., 2021). Fully-immunized for age is defined as the child having received all recommended national immunization schedule vaccines by the recommended age, adjusted for the current age of the child at the time of their inclusion in the NFHS-IV. Fair sources of variation in vaccination coverage include whether the child is underage to receive a vaccine according to India's national immunization schedule (National Health Mission, 2021). Due to the construction of the fully-immunized for age variable, it is impossible to be underage. As such, there is no fair source of variation that appears in the visual graphics. For zero-dose, the underage variable highlights the share of children under 24 months of age who have not received one or more recommended vaccines. These children are kept in the decomposition visualization as they are at risk of remaining zero-dose but are not included in the final equity outcome or the ranking procedure.

Unfair sources of variation included in the composite disadvantage ranking procedure include sex of the child, maternal education level, wealth index quintile, health insurance coverage, state of residence, and urban/rural designation. The direct unfairness healthcare ranking metric is assessed as the predicted probability of zero-dose vaccination status, holding the fair determinants at reference levels and allowing the unfair determinants to vary. This metric is then utilized as the ranking variable in a concentration index alongside vaccination coverage to compute the composite coverage concentration index. The multivariate concentration index is then decomposed to determine the percent contribution of each determinant to overall inequity and is presented alongside the AEG in coverage between the top and bottom quintile, based on composite disadvantage.

The overall concentration index for the multivariate equity measure is 0.404 (SE: 0.017) with an AEG of 0.120 (SE: 0.003) for zero-dose status, indicating that those with higher levels of unfair advantage are statistically significantly more likely to be vaccinated than those with lower levels of unfair advantage. Zero-dose prevalence among the most disadvantaged 20% of the population would need to decrease by approximately 12 percentage points to reach levels of the most advantaged 20%.

**Fig. 3:**
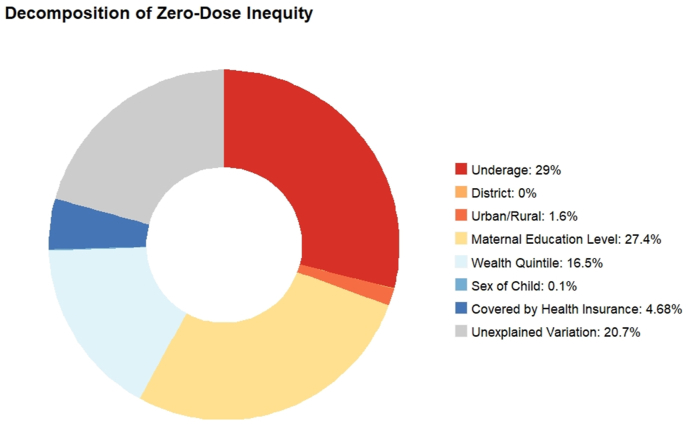
Decomposition of inequality in zero-dose status.

The primary correlate of unfair advantage in not having a zero-dose child is maternal education, accounting for 27.4% of the inequality in zero-dose status, followed by socioeconomic status, contributing 16.5%, and health insurance coverage, contributing 4.7%. Fair determinants of healthcare, being underage for zero-dose consideration by definition or too young to receive the first dose of DTP, OPV/IPV, or MCV vaccines at the time of the survey, according to the national immunization schedule, accounts for 29% of the overall inequity in zero-dose status (Fig. 3).

**Fig. 4:**
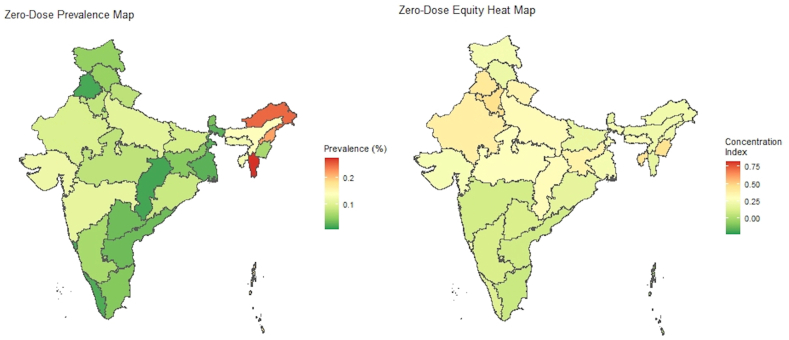
Zero-dose status prevalence & equity heat maps from the VERSE toolkit.

For Zero-Dose status, Arunachal Pradesh, Nagaland, Mizoram, Uttar Pradesh, Assam, Tripura, Meghalaya, and Gujarat rank among the top states in terms of Zero-Dose prevalence. Leaders in the inequality in zero-dose status due to unfair determinants are Punjab, Manipur, Chhattisgarh, Haryana, and Tripura (Fig. 4). Differences in state rankings are the result of how the possession of multiple unfair characteristics translates into actual vaccination status.

The overall concentration index for the multivariate equity measure is 0.140 (SE: 0.013) with an AEG of 0.371 (SE: 0.008) for fully-immunized for age, indicating that those with higher levels of unfair advantage are statistically significantly more likely to be vaccinated than those with lower levels of unfair advantage. As a result, full immunization prevalence among the most disadvantaged 20% of the population would need to increase by approximately 37.1 percentage points to reach levels of the most advantaged 20%.

**Fig. 5:**
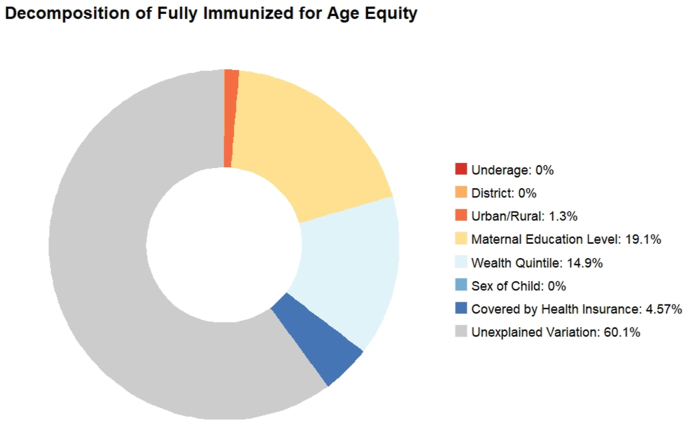
Decomposition of inequality in fully immunized for age.

The primary correlate of unfair disadvantage in achieving full immunization for age is, again, maternal education, accounting for 19.1% of the inequality in zero-dose status, followed by socioeconomic status, contributing 14.9%, and health insurance coverage contributing 4.6% (Fig. 5).

**Fig. 6:**
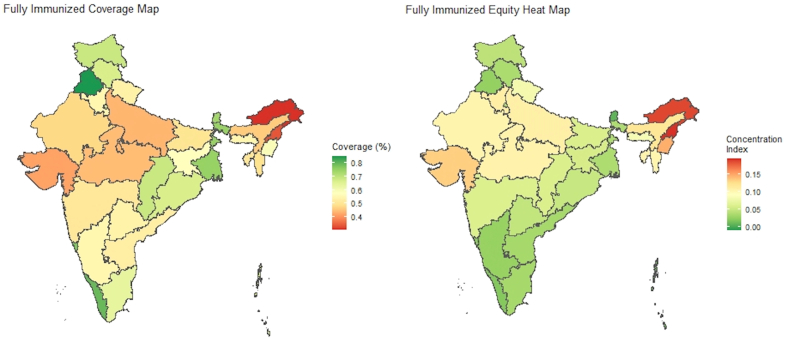
Fully-immunized for age prevalence & equity heat maps from the VERSE toolkit.

For being fully-immunized for age, Arunachal Pradesh, Nagaland, Mizoram, Uttar Pradesh, Assam, Tripura, Meghalaya, and Gujarat also rank among the states with the poorest performance in terms of achieving full immunization. Whereas zero-dose equity deviated from the prevalence map in terms of the relative ranking of states, fully-immunized for age inequality largely follows trends in prevalence, with Arunachal Pradesh, Nagaland, Tripura, Mizoram, Manipur, Uttar Pradesh, Gujarat, and Rajasthan having the least equal distribution of fully-immunized for age status, respectively (Fig. 6).

**Fig. 7:**
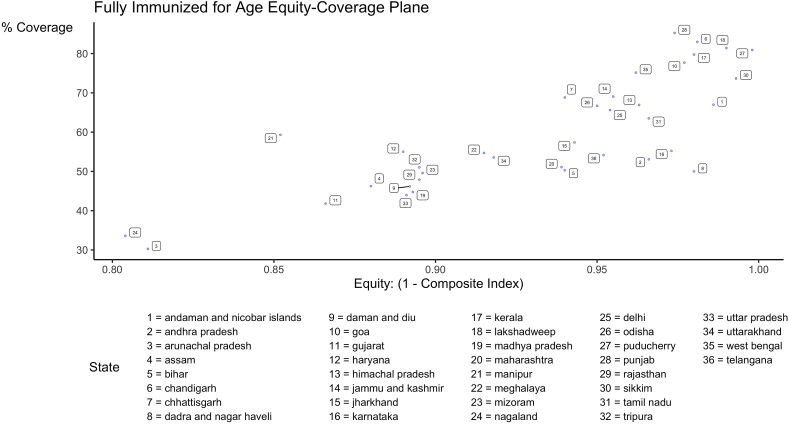
Equity-coverage plane for full immunization status from the VERSE toolkit.

Utilizing an equity-coverage plane, it is possible to compare the relative performance of states on both equity and coverage of all national immunization schedule vaccines used to compute fully-immunized status (Fig. 7). This is most useful for comparing states that achieved different levels of equity at comparable levels of immunization attainment. For example, the states of Manipur and Karnataka have attained near equivalent levels of full immunization coverage at 82.3% and 84%, respectively. However, Manipur lags behind Karnataka on the equity dimension with composite concentration indices of 0.079 and 0.011, respectively.

## 4 Discussion

The VERSE model builds upon bivariate equity metrics such as those assembled by the WHO on childhood immunization under the Health Equity Assessment Toolkit (HEAT) [3] by incorporating standardized methods for combining equity across multiple contributing factors into a single composite metric. The approach also permits expanding beyond equity in coverage to include equity in cost-of-illness, out-of-pocket expenditure, or modeled health outcomes related to vaccines in order to be more consistent with the multivariate framework for universal health coverage (UHC) (World Health Organization, 2021). The model is consistent with other forms of inequality metrics widely used in monitoring & evaluation and distributional analysis while also incorporating a standardized process for ranking individuals over disadvantage levels and incorporating fair and unfair contributing factors. This has critical applications to the health space for which disadvantage, in terms of access, is typically multivariate (Alonge and Peters, 2015; World Health Organization, 2018).

The case-study application to India demonstrates that, when examining multivariate inequality, maternal education contributes more toward relative inequality in vaccination coverage than socioeconomic status. This indicates that metrics only utilizing socioeconomic status as a basis for measuring inequality, in order to track whether or not access is pro-poor, miss a significant amount of variation in the overall equity in vaccination status (Wahl et al., 2021; Patenaude et al., 2021). While maternal education, socioeconomic status, and insurance coverage are significant contributors to overall inequality and explain over 50% of the total variation in being fully-immunized for age, they explain less than 50% of the variation in being zero-dose. This indicates that the baseline components of the VERSE model for multiple-disadvantage better account for the probability of attaining full immunization coverage than the probability of becoming zero-dose in India. More research is needed to explain the causal pathways between these contributing factors and zero-dose status and for filling the gaps to explain observed variation in vaccination status. Identifying additional routinely measured inputs could improve the relative ranking procedure and shed more light on the correlates of observed inequality.

Comparing the results with a recent publication on zero-dose status in India, we find near equivalent distribution in the prevalence in overall zero-dose status across states and comparable prevalence at the national level, despite defining zero-dose as never having received any routine immunization inclusive of DTP1, OPV1, and MCV1, instead of only examining DTP1 (Johri et al., 2021). The authors of that study also found higher concentrations of zero-dose status among those of rural populations, poorer wealth quintiles, and no maternal education with minimal differences between sex of the child. The decompositions of the inequality index conducted in this study help highlight that while urban/rural and state differences in zero-dose status are significant, the variation in inequality is almost entirely accounted for by maternal education and wealth, indicating that barriers to vaccine access may have more to do with poverty than geographic barriers. Additionally, the metric computed in this study can complement the work by Johri et al. by providing a single metric to compare overall progress toward reducing inequalities. For example, their study found that between 1998 and 2016, inequality between the poorest and richest quintiles declined drastically from a 41.6 percentage point difference in zero-dose prevalence in 1998 to an 8.9 percentage point difference in 2016 and the gap between the highest and lowest maternal education category decreased from 43.4 percentage points in 1998 to 11.8 percentage points in 2016, but the relative contribution of these two decreases to overall inequality is unknown. The VERSE model's composite concentration index could help to complement this data and show how composite inequality changed over time, as well as how the relative correlates of that inequality changed over time.

While the VERSE approach and toolkit can yield a stable metric to track equity over time or between settings, it is also subject to several practical limitations common to measures of inequality (Alonge and Peters, 2015). The first is the inability to objectively state what a “good” or “bad” level of inequality is. Like all concentration indices, the results of the VERSE methods lend themselves more toward assessing relative performance than to categorizing objective performance. Though values closer to 0 are objectively preferred, whether a value of 0.1 is bad or good depends upon the circumstances of a specific setting as well as benchmarks associated with the rollout of each vaccine. For this reason, all equity metrics should be put in the context of the outcome or intervention they are evaluating.

Additionally, the VERSE model produces an absolute equity gap alongside the concentration index to assist with interpretation. While the AEG is a measure of absolute inequality, and the concentration index measures relative inequality, they are both based on the same ranking procedure. They, therefore, can complement one another and help make the mathematical values digestible and meaningful for policy audiences. While the AEG can help to provide a more tangible absolute level difference for the observed inequality, the decompositions presented apply specifically to the concentration index metric.

Another limitation is the data requirements to populate the tool. Distributional equity indicators such as the VERSE composite metric rely on numerous low-level data points, such as individual-level survey data or neighborhood and community-level data. The data for the case study came from the NFHS-IV, a nationally representative survey collecting both socioeconomic, demographic, and health data (Government of India MOH Family and Welfare, 2017). However, not all locations have regularly collected representative survey data, which combines data across the multiple necessary dimensions to compute the composite equity metric. Despite this limitation, most Demographic and Health Surveys (DHS) do contain all necessary information to compute the base model of the VERSE composite metric for included vaccines. Additionally, many high-income countries have neighborhood-level data that could be used to compute the composite equity metric. However, stakeholders looking for more frequent assessments may be limited by data collected with greater frequency within a specific setting. Additionally, while the tool was designed to work alongside nationally-representative data and the case study presented is an application utilizing nationally-representative survey data, the methodologies are also applicable to surveys conducted within specific populations. The resulting interpretation would be internally valid to the population examined rather than nationally representative.

Finally, another limitation of this approach is that both the ranking procedure and the decomposition rely on correlations rather than causal inference. Therefore, while the decomposition sheds light on the overall level of inequity and the relative magnitude of contributing factors to variation in coverage, it does not speak to why those factors influence coverage or the causal pathway from the specific factor to coverage. The decompositions serve to provide a snapshot of the correlations with coverage, which should be complemented by deep-dive causal research to isolate the root cause of the associations observed in the decompositions.

## 5 Conclusions

Most equity analyses in healthcare only examine bivariate inequity or the decomposition of bivariate inequity into multiple dimensions. The VERSE model and toolkit measures and decomposes multivariate inequity in vaccination status to allow for standardized measurement over time and between locations. As a result, the VERSE toolkit can allow evaluators and decision-makers to determine the relative importance of contributors to overall inequity in health outcomes for better targeting programs, tracking progress toward equity goals, and understanding the overall impact of policies targeting only specific dimensions of inequity. Data permitting, this framework can be expanded across multiple healthcare access outcomes, financial risk protection outcomes, and health achievement outcomes to generate a composite health equity metric that tracks equitable progress toward universal health coverage beyond the vaccine space. The methods can also be utilized alongside primary data collection efforts to monitor or evaluate the multivariate equity in access or outcomes within a specific population or as a result of a specific intervention such as the equitable rollout of COVID-19 vaccines within a population over time.

## Author contributions

**Bryan N. Patenaude** conceived and designed the study, designed and reviewed analyses, contributed to the interpretation of data and to the writing of the manuscript. **Deborah Odihi** assisted with analyses, contributed to the interpretation of data and to the writing of the manuscript. **Salin Sriudomporn** assisted with analyses, contributed to the interpretation of data and to the writing of the manuscript. **Joshua Mak** assisted with analyses, contributed to the interpretation of data and to the writing of the manuscript. **Elizabeth Watts** assisted with analyses, contributed to the interpretation of data and to the writing of the manuscript. **Gatien de Broucker** assisted with the design of the study, running of analyses, and contributed to the interpretation of data and to the writing of the manuscript.

## Role of funding source

The funding source for this work was the 10.13039/100000865Bill & Melinda Gates Foundation under award INV-003813. The funding source submitted feedback on the overall study design during the initial proposal stage, the funding source has no role in the collection, analysis, and interpretation of data; in the writing of the report; or in the decision to submit the paper for publication.

## Declaration of competing interest

Bryan N. Patenaude has no conflicts of interest to declare.

Deborah Odihi has no conflicts of interest to declare.

Salin Sriudomporn has no conflicts of interest to declare.

Joshua Mak has no conflicts of interest to declare.

Elizabeth Watts has no conflicts of interest to declare.

Gatien de Broucker has no conflicts of interest to declare.
